# What is the impact of rerouting a cancer diagnosis from emergency presentation to GP referral on resource use and survival? Evidence from a population-based study

**DOI:** 10.1186/s12885-018-4274-0

**Published:** 2018-04-06

**Authors:** Mauro Laudicella, Brendan Walsh, Elaine Burns, Paolo Li Donni, Peter C. Smith

**Affiliations:** 1 School of Health Sciences, City, University of London, EC1V 0HB, London, UK; 2 Social Research Division, Economic and Social Research Institute, Dublin, D02 K138 Ireland; 3 Department of Surgery and Cancer, Imperial College London, St Mary’s Campus, London, W2 1NY UK; 4 Economics Department, University of Palermo, Viale delle Scienze, 90128 Palermo, Italy; 5 Business School, Imperial College London, South Kensington Campus, London, SW7 2AZ UK

**Keywords:** Route to diagnosis, Emergency, Primary care, Hospital costs, Survival, Early diagnosis

## Abstract

**Background:**

Studies on alternative routes to diagnosis stimulated successful policy interventions reducing the number of emergency diagnoses and associated mortality risk. A dearth of evidence on the costs of such interventions might prevent new policies from achieving more ambitious targets.

**Methods:**

We conducted a retrospective cohort study on the population of colorectal (88,051), breast (90,387), prostate (96,219), and lung (97,696) cancer patients diagnosed after a GP referral or an emergency presentation and reported in the Cancer Registry of England. Resource use and survival were compared 1 year before and 5 years after diagnosis (3 years for lung), including the costs of GP referrals not converted into a positive diagnosis. Risk-adjusted statistical models were used to calculate the effect of rerouting patient’ diagnoses from emergency presentation to GP referral.

**Results:**

Rerouting a cancer diagnosis results in a relatively small additional costs to the National Health System against additional years of life saved to the patient. The cost per year of life saved is £6456 in colorectal, £1057 in breast, −£662 in prostate (savings), and £819 in lung cancer. Reducing the overall prevalence of emergency presentations to the level achieved by the 20% of Clinical Commissioning Groups with the lowest prevalence would result in £11,481,948 against 1863 years of life saved for Colorectal, £847,750 against 889 years for breast, −£943,434 (cost savings) against 1195 years for prostate, and £609,938 against 1011 years for lung cancer.

**Conclusion:**

Redirecting diagnoses from emergency presentation to GP referral appears an achievable target that can produce large benefits to patients against modest additional costs to the National Health System.

**Electronic supplementary material:**

The online version of this article (10.1186/s12885-018-4274-0) contains supplementary material, which is available to authorized users.

## Background

Patients diagnosed after an emergency presentation (EP) experience considerably poorer short term survival than following alternative routes to diagnosis (RTD), such as a general practitioner (GP) referral [1–3]. One in five cancers diagnosed in England occur after an EP [1, 4, 5]. A late stage diagnosis can be caused by delays in patient presentation, delays in primary care, delays between primary and secondary care, and delays in secondary care [6]. Although EP are more likely to be associated with atypical symptoms, opportunities for an earlier diagnosis via alternative routes can be found in a substantial share of EP patients [7]. Reducing the number of diagnoses occurring after an EP could result in a considerable improvement in short term cancer survival reducing the gap between England and similar high income countries.

Studies on the health outcomes associated with alternative routes to diagnosis have stimulated a number of policy interventions aiming at reducing the number of patients diagnosed via the emergency routes. Initiatives such as the National Awareness and Early Diagnosis Initiative (NAEDI) and GP referral guidance issued by the National Institute of Health and Care Excellence (NICE) promote the earlier diagnosis of cancer through increasing the public awareness of symptoms; and increasing diagnoses through primary care [8]. There is, however, a dearth of evidence on the costs of such interventions and the long term consequences for the use of health resources. This might prevent health policies from achieving more ambitious targets in improving patient safety and cancer survival.

The objective of our study is to estimate the effect of rerouting a cancer diagnosis from EP to GP referrals on the utilisation of health care resources. Our analysis identifies cancer sites with scope for achieving better survival outcomes with a relatively small additional investment of resources. We also disentangled the effect on resource use that is driven by differences in survivals, i.e. patients living longer after their diagnosis, from the effect that is driven by differences in intensity of resource use, i.e. patients accessing more expensive treatments after their diagnosis. In the short term, EP patients are likely to use more resources for their treatment than patients diagnosed via a GP referral. However, the former are also likely to experience shorter survival times and have fewer opportunities to use health resources in the long term. Disentangling the intensity effect from the survival effect may be useful to assess the impact of policy interventions. Cost savings from reducing the intensity of resource use could be off-set by additional costs from extending the life of the patient. Therefore, a policy intervention could have little impact on total cost and yet achieve a desirable reallocation of resource use from intensity to survival.

## Methods

We performed an observational study on the population of patients with colorectal, breast, prostate and lung cancer. Patient-level data from each cohort are analysed separately using statistical models that allow for comparing utilisation of care in patients diagnosed via an EP and a GP after controlling for differences in patient characteristics. Patients are followed retrospectively 1 year before and up to 5 years after the diagnosis. Resource use before the diagnosis is likely to be driven by the cancer condition especially in patients with a late stage diagnosis who are more likely to follow the emergency route [1]. Therefore, including resource use before the diagnosis allows us to make a balanced comparison of costs across different routes. We also included the costs of non-converted cases (i.e. negative diagnosis) in the analysis. In order to achieve one extra diagnosis via a GP referral, a large number of patients with suspected cancer has to be referred for diagnostic tests. Therefore, the cost of the negative diagnoses should be added to the cost of the positive.

### Data

Our analysis uses a linked dataset including patient-level information from the National Cancer Data Repository (NCDR) matched with data on utilisation of inpatient and outpatient services from the Hospital Episodes Statistics (HES) and their costs from the National Schedules of Reference Costs (NSRC). NDCR matched to HES data were provided by the National Cancer Registration and Analysis Service (NCRAS) of Public Health England (PHE). The NCDR provides information on the characteristics of the patients, including age, tumour site, income deprivation in the area of residence, date of diagnosis, and date of death. The algorithm developed by Elliss-Brookes et al. [1] identifies patients diagnosed after an emergency presentation and after a GP referral in the dataset. We combined non-urgent and urgent 2 week wait (TWW) referrals in the analysis and referred to this route as GP/TWW referral throughout.

The HES database records information on patients’ utilisation of inpatient and outpatient hospital care for all National Health Service (NHS) patients in England, including information on principal and secondary diagnoses, operations performed, and method of admission. The NSRC includes information on the cost of inpatient and outpatient services accessed by NHS patients. Cost data are disaggregated at the level of Healthcare Resource Group (HRG) making special adjustments for patients’ type of admission, length of stay, and access to special services. A more detailed description of the procedure followed to match HES with NSRC data has been provided elsewhere [9]. The use of HES and NCDR for estimating inpatient costs has been validated in a previous study [10].

NCRAS provided estimates of the conversion rates from their analysis of the TWW referral data for the fiscal year 2014/15. Conversion rates are defined as the share of positive cancer diagnoses out of the total TWW referrals made in the year. Costs of non-conversion cases are obtained from a report study by Frontier Economics for the Department of Health [11].

The share of diagnoses occurring after an EP in the Clinical Commissioning Groups (CCGs) of England was calculated by using Cancer Registry data on patients diagnosed between January 2010 and December 2013.

### Patients

We included individuals aged over 18 with a diagnosis of colorectal cancer (ICD-10 code: C18, C19, C20), breast cancer (C50), prostate cancer (C61), or lung cancer (C33, C34) occurred after an EP or a GP/TWW referral and was reported in the cancer registries of England between March 2006 and April 2009. Cancer diagnoses occurring before 2006 were excluded as information on route to diagnosis is not available. We excluded individuals with a previous diagnosis of cancer, males with breast cancer, and patients reported to have died with improper death certificate (DCO) registrations.

Patients were followed up 1 year before and up to 5 years after their diagnosis until March 2010. We excluded patients diagnosed between April 2009 and March 2010 to allow for a minimum of 12 months follow-up for all patients.

### Endpoints

The primary outcome of this study is the utilisation of health resources, which was defined in terms of the cost of the care provided to patients by NHS hospitals. The terms “resource use” and “costs” are used interchangeably throughout the analysis. Resource use was measured at 2010 prices. Secondary outcome measures are 1 year and 5 years survival after diagnosis.

### Control variables

The statistical models used in the analysis include a number of control variables to allow for differences in patient characteristics that might influence resource use and survival outcomes between the EP and GP/TWW referral routes to diagnosis. We controlled for patients’ age at diagnosis, gender, and a set of comorbidity indicators based on the Charlson index, including: acute myocardial infarction, congestive heart failure, peripheral vascular disease, cerebrovascular disease, dementia, chronic obstructive pulmonary disease, rheumatoid disease, peptic ulcer, liver disease, diabetes, and renal disease. We also controlled for number of co-diagnoses reported during the first inpatient stay, a binary indicator if surgery performed in the first 12 months from diagnosis, and geographical region of residence, and income deprivation in the patient small area of residence (i.e., Lower Layer Super Output Area with average population of 1500 units).

Cancer staging was not used as a control variable as we aim to capture the impact of late and early diagnosis via the EP and the GP/TWW referral route.

### Statistical analysis

Estimating patient-level costs is challenging due to right censoring, and accelerated cost accumulation at the point of diagnosis and at the end of life. Cancer patients enter our study throughout the time period as they are diagnosed, between 2006 and 2009; hence resource use cannot be measured for the full 5 years for all patients, resulting in censored observations. The cost distribution is characterised by a U-shape with spikes in resource use at the time of diagnosis and at the end of life, which complicate the correct estimation of the effect of routes to diagnosis on costs [9, 12, 13]. Routes to diagnosis are likely to have an impact on costs through two distinct channels: by accelerating the use of resources (intensity effect) and by extending the life of the patient (survival effect). EP patients are more likely to use expensive resources over a short period of time after their diagnosis, while GP/TWW patients are more likely to use less expensive resources over a long period of time as they are more likely to survive cancer.

We estimated the effect of route to diagnosis on costs using the Basu-Manning (BM) estimator that allows for the comparison of retrospective patterns of utilisation of care in patients diagnosed after an EP and a GP referral addressing all the issues described above [14]. The BM-estimator consists in a three-part statistical model: a Probit model estimating differences in the probability of surviving and two Generalised Linear Models (GLM) estimating cost differences in every interval of time. The BM estimator assumes random censoring, which is a standard assumption in a wide class of survival models for time to event studies [14]. A detailed description of the BM estimator is included in a technical appendix file [see Additional file 1].

### Survival

Survival outcomes were examined by using continuous Weibull accelerated failure time models, which allows for estimating the survival outcomes associated with alternative routes after controlling for differences in patient characteristics described in the Control Variables section. In sensitivity analysis we found very similar predictions when using semi-parametric Cox regression models.

## Results

In total, 88,051 colorectal, 90,387 breast, 96,219 prostate, and 97,696 lung cancer patients were included. Table 1 shows the characteristics of patients in our study population by tumour sites. EP patients are older, have a higher morbidities and mortality risk as measured by the Weighted Charlson Index, and are more likely to come from an income deprived area across all tumour sites examined than the GP/TWW referrals. EP patients are less likely to receive surgery within 12 months of diagnosis than GP/TWW patients.

**Table 1:** Study population descriptive statistics

Tumour	Route to diagnosis	Number	Age	Female	IMD Income Deprivation (*)	Surgery within 12 Months (**)	Weighted Charlson Index	Surviving up to 12 Months
Colorectal	Emergency	27,729	73.83	45.68%	14.89%	38.16%	3.8	49.66%
	GP/TWW Referral	60,322	71.09	41.22%	12.94%	63.95%	2.62	78.71%
Breast	Emergency	6059	75.61	All	15.09%	15.28%	3.94	53.90%
	GP/TWW Referral	84,328	63.18	All	11.88%	73.94%	3.18	94.52%
Prostate	Emergency	10,350	77.89	None	14.22%	2.40%	3.7	59.15%
	GP/TWW Referral	85,869	70.88	None	12.47%	9.77%	2.29	94.91%
Lung	Emergency	43,026	73.81	44.15%	17.48%	1.99%	4.1	13.41%
	GP/TWW Referral	54,670	70.69	43.36%	15.97%	12.66%	3.29	39.77%

(*) IMD Income deprivation measures the share of people relying on mean tested income benefits in the patient area of residence

(**) OPCS 4.3 codes for surgery: H04, H05, H06, H07, H08, H09, H10, H11, H33 Colorectal; B27, B28 Breast; M611, M614, M618, M619 Prostate; E391, E398, E399, E441, E461, E541, E542, E543, E544, E545, E548, E549, E552, E559, E554, E574, E578, E595, T013 Lung

**Fig. 1:**
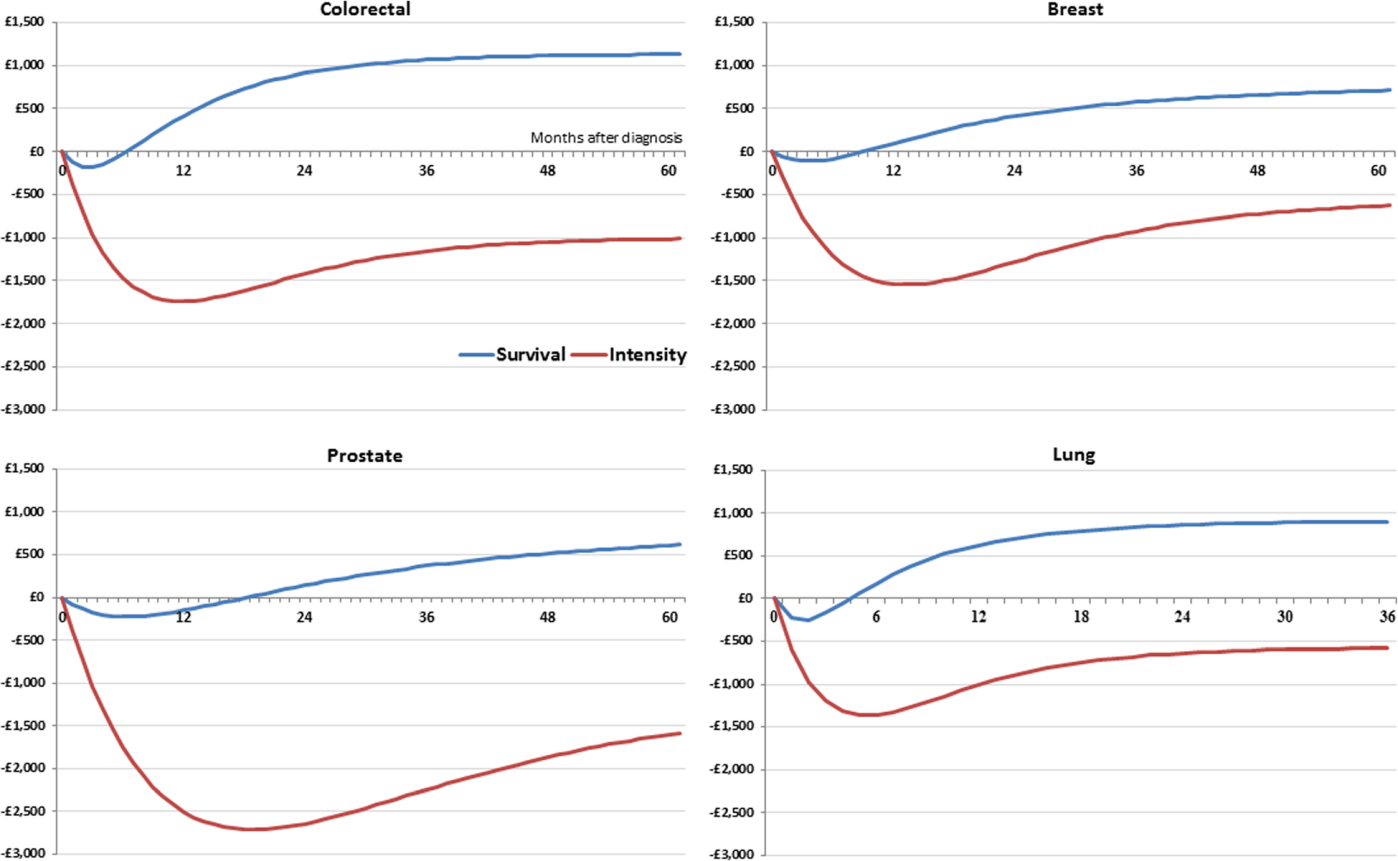
Cumulative Differences in Costs per Patient between GP/Two Week Wait Referral and Emergency Presentation (baseline). Differences in Costs Are Decomposed by Survival Effect (Blue line) and Intensity of Resource Use Effect (Red line). Risk-adjusted estimates are obtained from the Basu-Manning estimator. Negative values indicate GP/TWW referral is cost saving

Figure 1 plots the average effect on resource use of rerouting a patient from EP to GP/TWW referral. Figure 1 includes inpatient and outpatient costs only, and is cumulated by month from diagnosis to 5 year later (3 years for lung). Non-conversion costs and pre-diagnosis costs are considered in a separate analysis. Estimates are obtained from the BM estimator after controlling for patient characteristics described in the Control Variables section. Estimates can be interpreted as the increment (or reduction) in the use of inpatient and outpatient care associated with moving a patient from EP (the baseline) to GP/TWW referral. The effect plotted in Fig. 1 is decomposed in two parts: a part driven by differences in intensity of resource use and a part driven by differences in survivals between patients in the EP and GP/TWW route.

Rerouting patients from EP to GP/TWW referral results in a gradual increment in costs over 6 years due to better patient survival in all cancer cohorts, i.e. GP/TWW patients survive longer and have more opportunities to use resources in the long term. The rapid drop in costs in the first year from diagnoses is due to a reduction in intensity of resource use in all cohorts, i.e. GP/TWW patients consume less expensive resources per unit of time. Savings from the intensity effect gradually cancelled out after 5 years as the survival effect catches up with the intensity effect over time.

Table 2 shows the complete results of the cost analysis, including costs due to intensity of resource use, costs due to survivals, costs of resources used in the year before the clinical diagnosis, and costs of non-conversion cases, i.e. negative tests for patients referred for suspected caner via the GP/TWW route. Total costs per patient surviving up to 5 year are £25,716 for colorectal, £3179 for breast, −£2013 (savings) for prostate, and £3244 for lung cancer (3 years only). The costs per year of life saved up to 5 years post diagnosis are £6456 in colorectal, £1057 in breast, −£662 in prostate, and £819 in lung cancer (3 years only). It is worth noting that the largest share of the total cost of rerouting patients is absorbed by the non-conversion costs in all examined cancer cohorts.

**Table 2:** Effects of rerouting a cancer diagnosis from EP to GP/TWW referral

	Colorectal	Breast	Prostate	Lung
Conversion Rate (2014/15)	0.043	0.078	0.153	0.186
Costs per diagnostic test	£420	£158	£157	£127
Costs per converted case (a)	£9242	£1883	£874	£559
Variation in Costs 1 year pre diagnosis (b)	-£546	-£497	-£887	-£534
Variation in Survival Costs 1 year post diagnosis (c)	£479	£128	-£128	£669
Variation in Intensity Costs 1 year post diagnosis (d)	-£1734	-£1546	-£2565	-£952
Total Costs 1 year post diagnosis (a + b + c + d)	£7442	-£32	-£2706	-£258
Variation in Survival Costs 5 year post diagnosis (C)	£1128	£712	£616	£907
Variation in Intensity Costs 5 year post diagnosis (D)	-£1012	-£628	-£1582	-£577
Total Costs 5 year post diagnosis (a + b + C + D)	£8812	£1469	-£979	£356
Variation in Probability of Surviving up to 5 year	0.359	0.512	0.408	0.149
Total Cost per patient Surviving up to 5 year	£24,546	£2870	-£2399	£2388
Years of life saved up to 5 years post diagnosis (e)	1.43	1.54	1.24	0.59
Total Cost per year of life saved (e)	£6162	£954	-£789	£603

(*)Lung cancer patients are followed up to 3 years post diagnosis only

(a) Estimates from Frontier Economics for the Department of Health (2011)

(b) Risk-adjusted estimates from GLM model

(c)(d)(C)(D) Risk-adjusted estimates from the BM estimator

(e) Risk-adjusted estimates from Weibull models

**Fig. 2:**
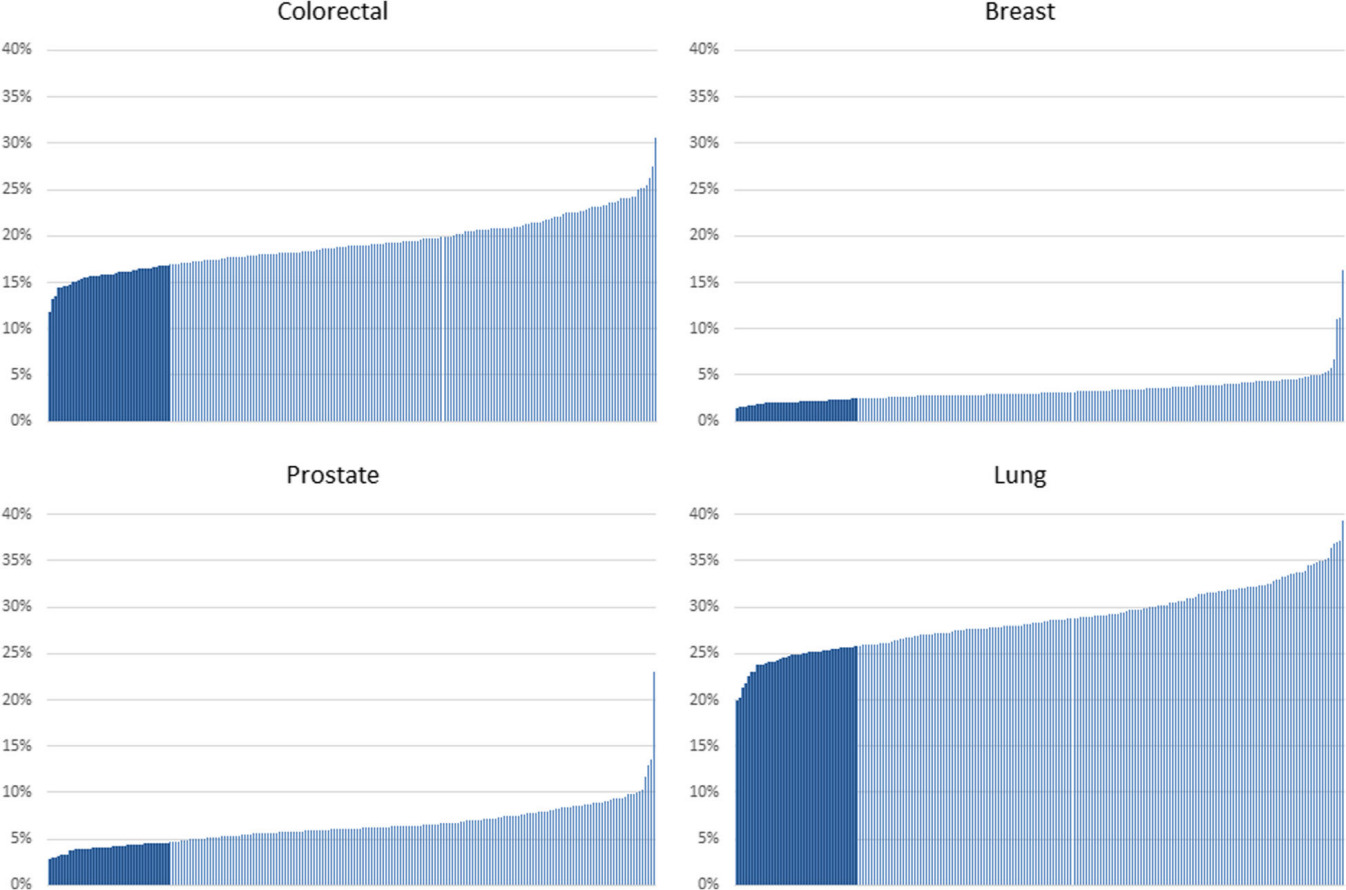
Share of Total Cancer Diagnoses Occurring after an Emergency Presentation in CCGs, 2010–2013. Each bar represents a CCG. Dark blue = Best performing 20% of CCGs; Light blue = Other CCGs

Figure 2 shows the share of EP diagnoses over the total cancer diagnoses made via Non-EP routes across all CCGs in England between 2010 and 2013. Figure 2 highlights a noticeable gap between the top performing 20% of CCGs with the lowest share of EP diagnoses (dark blue) and the rest of the CCGs (light blue).

Table 3 includes estimates of the potential impact of a policy reducing the share of EP diagnoses in all CCGs to match the average level of the top 20% of CCGs with the lowest share. The excess of EP diagnosis are rerouted to GP/TWW referrals. For simplicity we have assumed that achieving the policy target has no other costs than the direct medical costs included in Table 2, and we consider the long term effects after achieving the target for 1 year only. Achieving the policy target would result in rerouting 1303 diagnoses for colorectal, 577 for breast, 964 for prostate, and 1714 for lung in a year. They represent 3.16% of all new diagnoses for colorectal in a year, 1.04% for breast, 2.07% for prostate, and 3.69% for lung. In order to achieve these new diagnoses from the GP/TWW route, a much larger number of patients have to be referred for suspected cancer: 29,977 patients for colorectal, 7436 for breast, 6320 for prostate, and 1714 for lung. Non-conversions are the largest costs associated with the policy target as reported in Table 3. Total costs after 5 years would amount to £11.4 million for colorectal, £0.8 million for breast, and £0.6 million for lung cancer (3 year costs), resulting in 1863 years of life saved for colorectal, 889 for breast, 1195 for prostate and 1011 for lung cancer. Colorectal cancer costs dwarf other cancers due to the lower conversion rates and higher costs per non-conversion case as shown in Table 2. In contrast, achieving the policy target for prostate cancer would result in − 0.9 million of cost savings due to large intensity of care costs that would be avoided and the relatively small additional survival costs that would be incurred. The latter is explained by the small difference in survival costs between the EP and the GP/TWW route in prostate cancer relative to other cancers as illustrated in Fig. 1 and Table 2.

**Table 3:** Effects of decreasing the share of cancers diagnosed after an EP to match the average level achieved by the top 20% of CCGs with the lowest share

	Colorectal	Breast	Prostate	Lung
Patients rerouted from EP to GP/TWW referral	1303	577	964	1714
Non converted cases	29,977	7436	6320	9236
Total Costs 1 year post diagnosis	£9,696,503	-£18,260	-£2,608,444	-£441,712
Total Costs 5 year post diagnosis	£11,481,948	£847,750	-£943,434	£609,938
Patients surviving up to 5 years post diagnosis	468	295	393	255
Years of Life saved up to 5 years post diagnosis	1863	889	1195	1011

## Discussion

This study examined the impact on utilisation of health resources and survival of rerouting patients’ diagnosis from EP to GP/TWW referral. We examined a large population of colorectal (88,051), breast (90,387), prostate (96,219), and lung (97,696) cancer patients and measured resource use in terms of the cost of care to the NHS hospitals. The BM estimator is implemented to estimate patient-level costs with continuous censoring time and deaths, and to decompose the effect of routes to diagnosis on costs into the effect due to the intensity of resource use, i.e. patients consuming more resources per unit of time, and the effect due to survival, i.e. patients living longer and having more chances to access health resources. Our estimates show a modest cost per additional year of life saved in the 5 years following the diagnosis: £6456 in colorectal, £1057 in breast, −£662 in prostate (cost savings), and £819 in lung cancer (3 years only).

We also examined the potential effects of reducing the share of diagnoses occurring after an EP to match the average level in the top 20% of CCGs with the lowest share as observed during 2010–2013. We estimate the total impact of this policy target after 5 years is £11,481,948 against 1863 years of life saved for Colorectal, £847,750 against 889 years of life saved for breast, −£943,434 (cost savings) against 1195 years of life saved for prostate, and £609,938 against 1011 years of life saved for lung cancer (after 3 years). These estimates echo Abdel-Rahman et al. [15] study showing that 10,000 more patients would be alive at 5 years post-diagnosis if survivals in England matched the best in Europe.

Our findings have important policy implications. We provide evidence that policy interventions aimed at rerouting patients from EP to GP/TWW referral are likely to have a modest net impact in terms of additional costs for the NHS, while they are likely to result in large benefits to the patients. In the short term (1 year post diagnosis), rerouting patients’ diagnosis generates net savings in breast, prostate, and lung cancer, as additional costs due to non-conversions and improved survivals are outweighed by reduced costs due to lower intensity of resource use and pre-diagnosis costs. In the long term (5 years post diagnosis), costs due to improved survival increase and outweigh the initial savings. Prostate cancer is the only cohort showing savings after 5 years. Health policies aiming at reducing the number of diagnoses occurring after EP should be supported by appropriate resources to take care of a larger number of patients surviving cancer in the long term.

Moreover, we found evidence that a largest impact on costs is generated by non-conversion cases. As more patients are referred by GPs for suspected cancer, the conversion rate is likely to fall with a negative impact on costs. Therefore, clinical research on more effective and less expensive diagnostic tests will be key in supporting future policy interventions aiming at rerouting patients away from EP and late diagnoses. This is especially evident in colorectal cancer patients for whom the costs of colonoscopy testing are very high, and alternative diagnostic tools, such as CT colonography, can achieve large cost savings [16].

Our finding supports recent policy initiatives such as the National Awareness and Early Diagnosis Initiative (NAEDI), the Be Clear on Cancer campaign, and “Improving Outcomes: A Strategy for Cancer” [17] that have been successful in reducing the share of patients diagnosed via EP from 24% in 2006 down to 20% in 2013 [18].

### Existing evidence

A number of early studies investigated the effect of increasing the number of early diagnoses on costs using simulation modelling approaches [11, 19–22]. Our evidence is in line with Hinde et al. (2015) and Frontier Economics’ findings that increasing the number of early diagnoses is associated with a substantial increment in costs due to non-conversions and a small reduction in costs due to less intensive treatments for patients with a positive diagnosis. However, our evidence are not directly comparable with previous studies, since the starting point of our analysis is a shift in the distribution of the route to diagnosis, rather than in the distribution of cancer staging at diagnosis. Moreover, our observational study design presents a number of important differences from studies based on simulation models. First, our data allow for comparing resource use associated with different pathways of care and routes to diagnosis, without having to model patients’ transitions into different states of the disease that are often unobservable. Second, our analysis does not rely on evidence from clinical trials and expert opinions from clinical advisors on the “typical” pathways of care for patients in different states of the disease. On the one hand, our analysis does not allow for detailed costing of the different services that are likely to be accessed by patients, and for selecting only those services that have a direct link with the cancer treatment. On the other hand, it does allow for the inclusion of the costs of care generated by patients outside the typical pathway of care. Deviations from the pathway might occur for many different reasons, such as the patient’s personal circumstances (e.g. socioeconomic deprivation or living alone), the organisation of the local health services, and the medical practice at the treatment hospital. Observations on deviations from the typical pathways of care are key to determine EP costs, since diagnoses after an EP are likely to be the result of such deviations. Finally, our analysis allows for the indirect costs of cancer, i.e. costs generated as a result of the interactions between cancer and post and pre-existing health conditions. For instance, a patient with a chronic condition that normally can be managed in primary care might need a higher level of care to treat the same condition associated with cancer (i.e., multi-morbidity effect).

### Study limitations

Our cost estimates are based on a number of assumptions. We assumed GP referral conversion rates equal to conversion rates for TWW GP referrals as no data is available on the former. We assumed average diagnostic costs reported by other studies [11]. We considered pre-diagnosis costs 1 year before the diagnosis. We assumed that reducing the share of EP to the average share in the best performing 20% of CCGs is an achievable target. Each of the assumptions above is clearly listed in Tables 2 and 3, and their impact on estimated total costs can be easily assessed by replacing them with different values.

Patients diagnosed after an EP might be more likely to have fast growing and difficult to detect tumours as compared with patients diagnosed after a GP referral reducing the scope for changing the route to diagnosis [23]. We are unable to provide direct control for this potential confounding factor in our analysis. However, we observe a large variation in the share of patients detected after an EP across CCG geographical areas (Fig. 2), which we interpret as evidence that prevalence of fast growing cancer is unlikely to be the main driver and three is scope for reducing diagnostic delays.

Some patients who are diagnosed through an EP may not present to healthcare prior to their emergency admission limiting the scope for detection in primary care. We mitigated this problem by estimating the potential impact of matching the share of EP diagnoses observed in a large number of best performing CCG. This analysis assumed that there were no inherent differences in the propensity for patients to seek care in primary practice versus present as emergencies to secondary care. We did control for patients’ characteristics that are likely to increase the risk of diagnosis after EP, such as age, gender, deprivation, and chronic health conditions.

We considered five-year survival as the only health outcome in this study. However, some cancers suffer from lead-time bias, i.e. earlier diagnosis of disease might not necessarily improve outcomes, and survival does not always correspond to regression or cure of the tumour. Additionally, many cancers found through earlier diagnosis may be over diagnosed and would never have had any detrimental impact on patient’s health, thereby biasing estimated survivals. We mitigate such limitations by excluding patients with non-malignant tumours and excluding patients with history of malignant tumours.

Some of the tests performed on non-converted cases might lead to diagnosis for a different condition. Colonoscopy might detect an adenoma and potentially reducing longer term cancer risk. Therefore, part of the non-conversion costs might produce benefits that are not taken into account in our analysis. Nor does such analysis acknowledge the potential psychological impact on patients of increasing diagnostics for cancer.

Our study presents other limitations. Firstly, the analysis does not include the costs of primary care and social care. However, existing evidence shows that primary care absorbs a small share of total cancer costs, about 3% of total costs [24], while only a small share of patients use social care services after their diagnosis, 15% of lung, 13% of colorectal and 5% of breast and prostate cancer patients [25]. Secondly, the quality of cost information varies across hospitals and over time. To mitigate variation, we used costs at a fixed point in time (2010), excluded outliers, and calculated costs of similar services reported by different hospitals. Finally, the routes to diagnosis are identified using the algorithm formulated by Elliss-Brookes et al. [1] and related assumptions. In particular, for emergency presentation, all hospital activity in the 6 months pre-diagnosis was assumed to be due to the cancer; other authors consider a shorter cut-off point of 1 month [5].

## Conclusion

Policies aimed at increasing the proportion of patients diagnosed through primary care referral in colorectal, lung, breast and prostate cancer are likely to be cost-effective. Additional resources might be needed to support patients surviving longer after diagnosis. Improving the national rates in line with those achieved by the highest performing CCGs would result in a significant increase in diagnostic tests for suspected cancer and associated costs.

## Additional file

Additional file 1: Technical Appendix. Description of the Basu-Manning estimator used in the statistical analysis. (DOCX 23 kb)

## References

[1] Elliss-Brookes L, McPhail S, Ives A, Greenslade M, Shelton J, Hiom S (2012). Routes to diagnosis for cancer - determining the patient journey using multiple routine data sets. Br J Cancer.

[2] McPhail S, Elliss-Brookes L, Shelton J, Ives A, Greenslade M, Vernon S (2013). Emergency presentation of cancer and short-term mortality. Br J Cancer.

[3] Sheringham JR, Georghiou T, Chitnis XA, Bardsley M (2014). Comparing primary and secondary health-care use between diagnostic routes before a colorectal cancer diagnosis: Cohort study using linked data. Br J Cancer.

[4] NCIN (2015). Routes to diagnosis 2006–2013 preliminary results short report [internet].

[5] Tsang C, Bottle A, Majeed A, Aylin P (2013). Cancer diagnosed by emergency admission in England: An observational study using the general practice research database. BMC Health Serv Res.

[6] Rubin G, McPhail S, Elliott K (2011). National Audit of Cancer diagnosis in primary care.

[7] Renzi C, Lyratzopoulos G, Card T, Chu TPC, Macleod U, Rachet B (2016). Do colorectal cancer patients diagnosed as an emergency differ from non-emergency patients in their consultation patterns and symptoms? A longitudinal data-linkage study in England. Br J Cancer.

[8] National Institute for Health and Care Excellence (2015). Suspected cancer: Recognition and referral [internet].

[9] Laudicella M, Walsh B, Burns E, Smith PC. Cost of care for cancer patients in England: Evidence from population-based patient-level data. Br J Cancer. 2016; [cited 2016 Apr 13]; Available from: http://www.nature.com/doifinder/10.1038/bjc.2016.77 10.1038/bjc.2016.77 PMC4891510 27070711

[10] Thorn JC, Turner EL, Hounsome L, Walsh E, Down L, Verne J (2016). Validating the use of hospital episode statistics data and comparison of costing methodologies for economic evaluation: An end-of-life case study from the cluster randomised triAl of PSA testing for prostate cancer (CAP). BMJ Open.

[11] DH & Frontier Economics (2011). The likely impact of earlier diagnosis of cancer on costs and benefits to the NHS.

[12] Brown ML, Riley GF, Schussler N, Etzioni R (2002). Estimating health care costs related to cancer treatment from SEER-Medicare data. Med Care.

[13] Yabroff KR, Lamont EB, Mariotto A, Warren JL, Topor M, Meekins A (2008). Cost of Care for Elderly Cancer Patients in the United States. J Natl Cancer Inst.

[14] Basu A, Manning WG (2010). Estimating lifetime or episode-of-illness costs under censoring. Health Econ.

[15] Abdel-Rahman M, Stockton D, Rachet B, Hakulinen T, Coleman MP (2009). What if cancer survival in Britain were the same as in Europe: How many deaths are avoidable?. Br J Cancer.

[16] Atkin W, Dadswell E, Wooldrage K, Kralj-Hans I, von Wagner C, Edwards R (2013). Computed tomographic colonography versus colonoscopy for investigation of patients with symptoms suggestive of colorectal cancer (SIGGAR): A multicentre randomised trial. Lancet.

[17] Department of Health (2011). Improving outcomes: A strategy for Cancer [internet].

[18] PHE (2015). Cancers are being diagnosed earlier in England - press releases - GOV.UK [internet].

[19] Hinde S, McKenna C, Whyte S, Peake MD, Callister MEJ, Rogers T (2015). Modelling the cost-effectiveness of public awareness campaigns for the early detection of non-small-cell lung cancer. Br J Cancer.

[20] Incisive Health (2014). Saving lives averting costs: An analysis of the financial implications of achieving earlier diagnosis of colorectal, lung and ovarian cancer - a report prepared for Cancer Research UK [internet].

[21] Bending MW, Trueman P, Lowson KV, Pilgrim H, Tappenden P, Chilcott J (2010). Estimating the direct costs of bowel cancer services provided by the National Health Service in England. Int J Technol Assess Health Care.

[22] Pilgrim H, Tappenden P, Chilcott J, Bending M, Trueman P, Shorthouse A (2009). The costs and benefits of bowel cancer service developments using discrete event simulation. J Oper Res Soc.

[23] Neal RD (2009). Do diagnostic delays in cancer matter?. Br J Cancer.

[24] Luengo-Fernandez R, Leal J, Gray A, Sullivan R (2013). Economic burden of cancer across the European Union: A population-based cost analysis. Lancet Oncol.

[25] Nuffield Trust (2014). Use of health and social care by people with cancer.

