# Supplementary material for: What is the impact of rerouting a cancer diagnosis from emergency presentation to GP referral on resource use and survival? Evidence from a population-based study
Source: BMC Cancer. 2018 Apr 6;18:394. doi: 10.1186/s12885-018-4274-0 (PMC5889525; doi:10.1186/s12885-018-4274-0)
Supplement: Supplementary file 1 — Technical Appendix. Description of the Basu-Manning estimator used in the statistical analysis. (DOCX 23 kb) [file 12885_2018_4274_MOESM1_ESM.docx]

Technical Appendix

The Basu and Manning (BM) estimator was used to model costs after diagnosis in our statistical analysis. The BM estimator extends the estimator proposed by [1] allowing for continuous death and censoring time and for of the decomposition of the exposure variable effect into an intensity effect and a survival effect. Right censoring is a key issue in modelling costs as patients can reach the diagnosis of cancer at any point in time between the beginning and the end of our study period. Therefore, resource use cannot be measured for the same period of time for all patients, and the statistical analyses have to be appropriately adjusted. Moreover, modelling patient-level costs is challenging as the cost distribution is typically U-shaped with high peaks in resource use at the point of diagnosis and at the end of life, and low use of resources in between [2–4].

The BM estimator consists of a three part model which allows for a great flexibility in modelling patient-level costs. We implemented the BM estimator following the application in [5]. We started with specifying the dataset at the patient-period level by selecting 30 days periods. We then estimated three separate models:

1. **Survival Model:** A survival model is estimated as a function of route to diagnosis and control variables using the patient-period observations in which the patients is not censored and is alive at the beginning of the period. The estimated coefficients from the survival model are used to predict the probability of survival until the start of the period and the hazard function for death during the period for all patient-period observations. We use a discrete-time pooled logistic regression model to estimate survival as in a previous application [5]; discrete-time model reduces computation time as it is easy to estimate using patient-period observations and allows for time-varying covariates effects. We find no difference comparing this model’s predictions with a continuous-time Cox proportional model.
2. **End-of-Life Costs Model:** a generalized linear model (GLM) with log link and gamma distribution is used to estimate end-of-life costs as a function of route to diagnosis and control variables using the patient-periods in which the patient dies, following previous applications [5,6]. This model allows for the skewness in the distribution of patient-level costs, which are not normally distributed as a large proportion of patients having high costs at the end of life. As the patient may die at any point in the 30-day period, the number of surviving days is also included as a control variable in the model. The model is then used to predict costs in all patient-periods, including patient-periods in which the patient does not die or is censored. This part of the BM estimator essentially allows for death to be modelled as a continuous-time event.
3. **Continuous-time Costs Model:** a third model is applied to estimate costs in the patient-periods where a person does not die and is not censored. Since patients may use no healthcare resource in a considerable number of periods, we use a two-part model to estimate costs [7]. The first part of the model is a logit that estimates the probability of using health resources, and the second part is a GLM with log link and gamma distribution that estimates total resource use conditioning on the probability of resource use to be greater than zero. The model is then used to predict costs in all patient-periods, including patient-periods in which the patient dies or is censored. This part of the BM estimator allows for modelling continuous censoring time.

The three models outlined above provide us with a predicted hazard function for each period$\left( \hat{h}_{j} \right)$, a predicted cumulative survival function for each period$\left( \hat{S}_{j} \right)$, and predicted costs in periods where a person dies$\left( \hat{\mu}_{1j} \right)$, and predicted costs in periods where a person does not die$\left( \hat{\mu}_{2j} \right)$, with *j* denoting the time period. The estimated cost function for the period *j* can be expressed as [6]:

| $\mu_{j}=S_{j}*\left[ h_{j}*\mu_{1j}+\left( 1-h_{j}*\mu_{2j} \right) \right]$ | (1) |
| --- | --- |

The average patient cost can be obtained by simply adding the costs in each period:

| $\mu=\sum_{j=1}^{k} \mu_{j}$ | (2) |
| --- | --- |

The effect of route to diagnosis on costs can be measured by calculating the average marginal effects (AME) from the BM models; AME can be interpreted as the average change in resource use that would occur if all patients were switched from one route to another. The AME can be calculated in every period and expressed as an incremental effect over time from diagnosis:

| $\frac{\Delta\mu}{\Delta R}=\sum_{j=1}^{k} \frac{\Delta\mu_{j}}{\Delta R}$ | (3) |
| --- | --- |

where $\frac{\Delta\mu}{\Delta R}$ is the difference in costs$(\Delta\mu)$ associated to a change in the route to diagnosis$(\Delta R)$.

Finally, the BM estimator allows for decomposing the incremental effect in (3) into an intensity effect and a survival effect. The former capture the effect on costs brought about by differences in the rate of cost accumulation between alternative routes to diagnosis, while the latter captures the effect on costs brought about by differences in survival [6]:

| $\frac{\Delta\mu}{\Delta R}=\sum_{j=1}^{K} \left[ \left\{ \frac{\Delta S_{j}}{\Delta R}\left[ h_{j}*\mu_{1j}+\left( 1-h_{j}+\mu_{2} \right)+S_{j}*\left[ \frac{\Delta h_{j}}{\Delta R}\left( \mu_{1j}-\mu_{2j} \right) \right] \right] \right\}+\left\{ S_{j}*\left[ h_{j}\frac{\Delta\mu_{1j}}{\Delta R}+\left( 1-h_{j} \right)*\frac{\Delta\mu_{2j}}{\Delta R} \right] \right\} \right]$ | (4) |
| --- | --- |

where the$\frac{\Delta S_{j}}{\Delta R}\left[ h_{j}*\mu_{1j}+\left( 1-h_{j}+\mu_{2} \right)+S_{j}*\left[ \frac{\Delta h_{j}}{\Delta R}\left( \mu_{1j}-\mu_{2j} \right) \right] \right]$ represents differences in costs due to the survival effect, while$S_{j}*\left[ h_{j}\frac{\Delta\mu_{1j}}{\Delta R}+\left( 1-h_{j} \right)*\frac{\Delta\mu_{2j}}{\Delta R} \right]$ represents the differences in costs due to the intensity effect.

References

1. Lin DY, Feuer EJ, Etzioni R, Wax Y. Estimating medical costs from incomplete follow-up data. Biometrics. 1997;53:419–34.

2. Brown ML, Riley GF, Schussler N, Etzioni R. Estimating health care costs related to cancer treatment from SEER-Medicare data. Med. Care. 2002;40:IV–104.

3. Yabroff KR, Lamont EB, Mariotto A, Warren JL, Topor M, Meekins A, et al. Cost of Care for Elderly Cancer Patients in the United States. JNCI J. Natl. Cancer Inst. 2008;100:630–41.

4. Laudicella M, Walsh B, Burns E, Smith PC. Cost of care for cancer patients in England: evidence from population-based patient-level data. Br. J. Cancer [Internet]. 2016 [cited 2016 Apr 13]; Available from: http://www.nature.com/doifinder/10.1038/bjc.2016.77

5. Federspiel JJ, Stearns SC, D’Arcy LP, Geissler KH, Beadles CA, Crespin DJ, et al. Resource Use Trajectories for Aged Medicare Beneficiaries with Complex Coronary Conditions. Health Serv. Res. 2013;48:753–72.

6. Basu A, Manning WG. Estimating lifetime or episode-of-illness costs under censoring. Health Econ. 2010;19:1010–28.

7. Mullahy J. Much ado about two: reconsidering retransformation and the two-part model in health econometrics. J. Health Econ. 1998;17:247–81.
